# Burn Admissions Across Low- and Middle-income Countries: A Repeated Cross-sectional Survey

**DOI:** 10.1093/jbcr/irac096

**Published:** 2022-07-08

**Authors:** Laura Quinn, Tanveer Ahmed, Henry Falk, Ariel Miranda Altamirano, Adelin Muganza, Kiran Nakarmi, Ahmed Nawar, Michael Peck, Shankar Man Rai, Jo Sartori, Luiz Philipe Molina Vana, Benjamin Wabwire, Naiem Moiemen, Richard Lilford

**Affiliations:** Institute of Applied Health Research, University of Birmingham, Edgbaston, UK; Sheikh Hasina National Institute of Burn and Plastic Surgery, Dhaka, Bangladesh; Department of Environmental Health, Rollins School of Public Health, Emory University, Atlanta, Georgia, USA; Pediatric Burn Unit, Hospital Civil de Guadalajara, Guadalajara, México; Department of Surgery, Chris Hani Baragwanath Academic Hospital, University of the Witwatersrand, Johannesburg, South Africa; Department of Burns, Plastic and Reconstructive Surgery, phect-NEPAL, Kirtipur Hospital, Kathmandu, Nepal; Plastic Surgery Department, Kasr Al-Ainy School of Medicine, Cairo University, Cairo, Egypt; University of Arizona College of Medicine, Phoenix, USA; Department of Burns, Plastic and Reconstructive Surgery, phect-NEPAL, Kirtipur Hospital, Kathmandu, Nepal; National Academy of Medical Science, Kathmandu, Nepal; Institute of Applied Health Research, University of Birmingham, Edgbaston, UK; Department of Plastic Surgery, Hospital das Clinicas HCFMUSP, Faculdade de Medicina, Universidade de Sao Paulo, Brazil; Kenyatta National Hospital, Nairobi, Kenya; College of Medical and Dental Sciences, University of Birmingham, Edgbaston, UK; University Hospital Birmingham NHS Foundation Trust, Queen Elizabeth Hospital Birmingham, Edgbaston, UK; Institute of Applied Health Research, University of Birmingham, Edgbaston, UK

## Abstract

Burn injuries have decreased markedly in high-income countries while the incidence of burns remains high in Low- and Middle-Income Countries (LMICs) where more than 90% of burns are thought to occur. However, the cause of burns in LMIC is poorly documented. The aim was to document the causes of severe burns and the changes over time. A cross-sectional survey was completed for 2014 and 2019 in eight burn centers across Africa, Asia, and Latin America: Cairo, Nairobi, Ibadan, Johannesburg, Dhaka, Kathmandu, Sao Paulo, and Guadalajara. The information summarised included demographics of burn patients, location, cause, and outcomes of burns. In total, 15,344 patients were admitted across all centers, 37% of burns were women and 36% of burns were children. Burns occurred mostly in household settings (43–79%). In Dhaka and Kathmandu, occupational burns were also common (32 and 43%, respectively). Hot liquid and flame burns were most common while electric burns were also common in Dhaka and Sao Paulo. The type of flame burns varies by center and year, in Dhaka, 77% resulted from solid fuel in 2014 while 74% of burns resulted from Liquefied Petroleum Gas in 2019. In Nairobi, a large proportion (32%) of burns were intentional self-harm or assault. The average length of stay in hospitals decreased from 2014 to 2019. The percentage of deaths ranged from 5% to 24%. Our data provide important information on the causes of severe burns which can provide guidance in how to approach the development of burn injury prevention programs in LMIC.

A burn is an injury to the skin or other tissue primarily caused by exposure to heat and hot substances. Burns are an important cause of death and survivors may be left with lifelong disabilities.^1^ The World Health Organisation (WHO) estimates that 11 million burn injuries occur each year, of which 180,000 lead to death.^2^ In high-income countries, there has been a large reduction in burn injuries. Burn injuries remain a massive issue in Low- and Middle-Income Countries (LMICs) and more than 90% of burns are thought to occur in these countries.^2^

The majority of burns are small and do not require hospital admissions. Information on burn epidemiology is inconsistent, with high-income countries having more available data compared to LMICs. Obtaining epidemiological data on burns is not easy. From a data collection perspective, burns can be divided into large burns requiring hospital specialising in burn injury and smaller burns that do not lead to hospital admission. Collecting data on the latter is difficult and must be approached either by collecting records from a huge array of health facilities or by population surveys.

Large burns cause the greatest mortality and loss of amenity. Such burns can be ascertained through the records of centers in hospitals catering for severe burn injuries. This is not an ideal epidemiological method, since the dominator population for a particular burns center is hard to define. Nevertheless, studies of hospital admissions enable the trends in the incidence of burns to be observed over time (assuming no radical change in referral patterns) and can track changes in causes of burn injuries over time and between countries.

This paper aims to summarise information on burn admissions from eight burn centers in LMICs in 2014 and 2019. This information includes the total number of burn admissions, demographics of burn patients, setting of burn occurrence, type and cause of burns, and outcomes of burn admissions.

## METHODS

### Study Design and Setting

This study design was a repeated cross-sectional survey. The observation periods cover the calendar years 2014 and 2019. In 2021, the local investigator at each participating burn center was asked to complete a survey based on the WHO global burn registry form from routinely collected data (see Supplementary Appendix 1).^3^ A sample of the centers was contacted regarding the availability of data in their burn databases before the data extraction forms were sent. Nevertheless, the information available in each center varied from center to center as some centers did not collect certain data points included in the survey.

Eight centers from different LMICs contributed data: Cairo (Egypt); Nairobi (Kenya); Ibadan (Nigeria); Johannesburg (South Africa); Dhaka (Bangladesh); Kathmandu (Nepal); Sao Paulo (Brazil), and Guadalajara (Mexico). The centers were chosen as they cover Africa, Asia, and Latin America, they experience high burn rates and we have contacts in each of the centers. Details on the geopolitical characteristics of the countries and information on the burn centers are given in Table 1.

**Table 1.** Geopolitical table of the eight counties from the World Bank data and center information

		Africa				Asia		Latin America	
Characteristic		Egypt	Kenya	Nigeria	South Africa	Bangladesh	Nepal	Brazil	Mexico
Country Information									
Income Classification (Lower/Upper-Middle)		Lower	Lower	Lower	Upper	Lower	Lower	Upper	Upper
Total Population (millions)	2014	90.4	46.7	176.4	54.5	154.5	26.9	202.8	120.4
	2019	100.4	52.6	201.0	58.6	163.1	28.6	211.1	127.6
GDP (US$ billion)	2014	305.6	68.3	546.7	381.2	172.9	22.7	2456.1	1315.4
	2019	303.1	100.6	448.1	387.9	302.6	34.2	1877.8	1269.4
Percentage Aged <15-years-old	2014	33.2	41.9	44.2	29.4	29.8	34.0	22.9	28.0
	2019	33.8	39.2	43.7	29.0	27.2	29.6	21.0	26.2
Rural Population (% Total Population)	2014	57.2	74.8	53.0	35.7	66.5	81.8	14.5	21.0
	2019	57.3	72.5	48.8	33.1	62.6	79.8	13.2	19.6
Center Information									
Location Burn Center		Cairo (urban)	Nairobi (urban)	Ibadan (urban)	Johannesburg (urban)	Dhaka (urban)	Kathmandu (urban)	Sao Paulo (urban)	Guadalajara (urban)

### Africa

Egypt has a population of around 100 million people, just more than half the population live in rural areas. Our participating center is the Kasr Al-Ainy burn unit in Cairo University hospital. It is one of the oldest and best-established burn center and serves Cairo and other governates. It has 21 beds with an intensive care facility and an Intensive Care Unit (ICU).^4^ There is another burn unit in Cairo in the Ain Shams University. Beyond this, we have little information on burn facilities in Cairo.

Kenya has a population of about 50 million people, more than 70% of people live in rural areas and about 40% are under 15-years-old. In Kenya, the main center is in the Kenyatta National Hospital in Nairobi, the leading referral hospital in the country with 100 beds for burn patients.^5^

Nigeria has a population of about 200 million people, around 50% live in rural areas and more than 40% are less than 15-years-old. Our participating center is in the University College Hospital in Ibadan which opened in 2011 and has a 12 bed ward.^6^

South Africa has a population of about 59 million, around 35% of people live in rural areas. Burns are a major problem due to the proportion of people living in poorly constructed, combustible accommodation in urban areas.^7^ In South Africa, the centers are an adult and pediatric burn units in Baragwanath Hospital in Johannesburg which is the largest hospital in Africa.^8^ The adult burn unit consists of 24 ward beds and six ICU beds^9^ while the pediatric burn unit has 24 ward beds and 8 ICU beds.^10^

### Asia

Bangladesh has a population of about 160 million, more than 60% live in rural areas. In Bangladesh, our participating center is the Sheikh Hasina National Institute of Burn and Plastic Surgery in Dhaka which is the largest burn and plastic surgical care center in the world with 500 beds, 50 ICU beds, and 12 operation theatres.^11^ This center is the only tertiary burn care center in Bangladesh and therefore, receives a wide range of patients from across the country.^12^ This new hospital replaced the previous burns unit but continued the same routine data collection system.

Nepal has a population of about 29 million and the majority of people live in rural areas. Nepal is a mountainous country with transportation and road access challengers. There are few facilities that provide organised burn care in Nepal. The Nepal Cleft and Burn Center, out participating center in this study is the only dedicated center in the country.^13^ The center is based in Kirtipur Hospital in Kathmandu which has 30 beds (7 ICU beds) and two dedicated burn operating rooms which run six days a week.^14^ The center received patients from all districts in Nepal and is nearly entirely dependent on donations.^15^

### Latin America

Brazil has a population of more than 200 million people, only 20% are under 15-years-old and about 14% live in rural areas. In Brazil, the participating center is in the Federal University of Sao Paulo or Sao Paulo hospital, one of six burns care units in public hospitals serving the municipality of Sao Paulo which has a population of almost 20 million^16^

Mexico has a population of about 120 million people, of which about 20% live in rural areas. In Mexico, the participating center is in the Guadalajara Civil Hospital. Most burn patients are hospitalised from the emergency department to the 14 bed ICU as it is the only facility for treating critically ill burn patients.^17^

### Data Collection

Local investigators were asked to extract data and summarise burn admissions for the calendar years 2014 and 2019. The month that burn admissions occurred and the number of admissions with a TBSA of more than 20% was recorded. Age groups were categorised as adults and children using the closest available cut-off point to 12 years of age. The setting in which burns occurred was categorised as household, occupational, other, or unknown and the location where the burn occurred was ategorized as urban (including peri-urban) or rural. The types of burns were ategorized as flame, hot surface, hot liquid, direct electric, chemical, other, or not known with further detail given on flame burns given when available. The cause of burns was categorised as unintentional, intentional self-harm, assault, or unknown. The outcomes of burn admissions were average length of hospital stay measured in days and the number of deaths that occurred.

### Statistical Methods

Demographics of burn admission patients, details of burns admissions such as setting and location, type of burns, and outcomes of burn admissions were summarised using counts and percentages for categorical data and means or medians for numeric data. All analysis was performed in Stata v16.1. No comparisons were conducted since this is a descriptive study, there is no well-defined denominator population and even incidence rates ratios could be affected by changes in denominator populations.

## RESULTS

### Total Admissions

**Figure 1.**
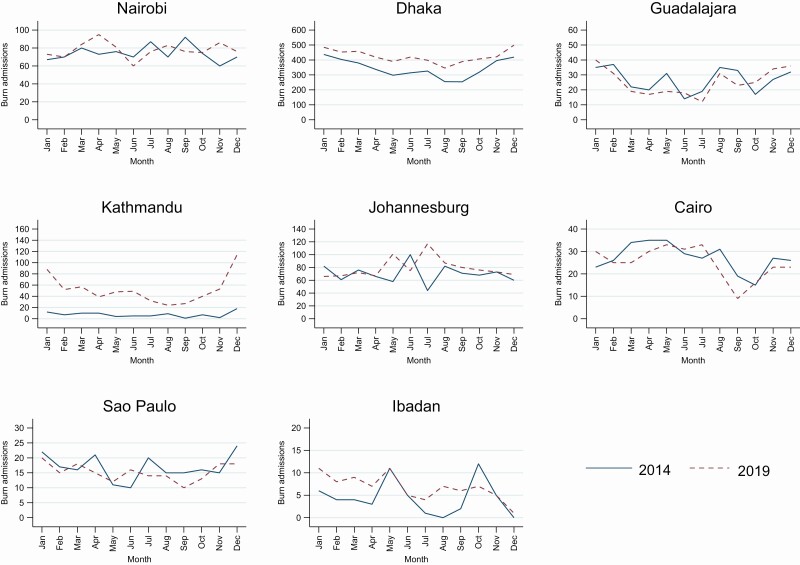
Burn admission by center, year, and month.

Across the eight burn centers, 15,344 patients were admitted in 2014 and 2019. The total burn admissions increased from 6907 patients in 2014 to 8437 patients in 2019. The largest increases in total admissions were in Dhaka and Kathmandu (Table 2, Figure 1). The burn admissions in Dhaka increased from 4139 admissions in 2014 to 5084 admissions in 2019. This increase could have resulted from the opening of the new hospital which likely simulated more referrals from around the country. In Kathmandu, the burn admissions increased from 90 admissions in 2014 to 623 admissions in 2019. For the other centers the number of burn admissions was similar in both time periods. In Dhaka and Kathmandu, burns admissions increase in the winter months (November to February).

**Table 2.** Burns admissions by center, year, and month

				Number of Admissions Per Month (%)												
Region	Center	Year	Total Admissions	Jan	Feb	Mar	Apr	May	Jun	Jul	Aug	Sept	Oct	Nov	Dec	NK
Africa	Cairo	2014	327	23 (7)	26 (8)	34 (10)	35 (11)	35 (11)	29 (9)	27 (8)	31 (9)	19 (6)	15 (5)	27 (8)	26 (8)	0
		2019	303	30 (10)	25 (8)	25 (8)	30 (10)	33 (11)	31 (10)	33 (11)	21 (7)	9 (3)	16 (5)	23 (8)	23 (8)	4 (1)
	Nairobi	2014	889	67 (8)	70 (8)	80 (9)	73 (8)	76 (9)	70 (8)	87 (10)	70 (8)	92 (10)	74 (8)	60 (7)	70 (8)	0
		2019	935	73 (8)	70 (7)	84 (9)	95 (10)	81 (9)	60 (6)	76 (8)	83 (9)	76 (8)	75 (8)	86 (9)	76 (8)	0
	Ibadan	2014	53	6 (3)	4 (2)	4 (2)	3 (1)	11 (5)	5 (2)	1 (0)	0	2 (1)	12 (6)	5 (2)	0	0
		2019	81	11 (6)	8 (4)	9 (5)	7 (4)	11 (6)	5 (3)	4 (2)	7 (4)	6 (3)	7 (4)	5 (3)	1 (1)	0
	Johannesburg	2014	882	82 (10)	61 (7)	76 (9)	66 (8)	58 (7)	100 (12)	44 (5)	82 (10)	71 (8)	68 (8)	73 (9)	60 (7)	0
		2019	909	66 (7)	67 (7)	72 (8)	67 (7)	101 (11)	75 (8)	117(12)	87 (9)	80 (8)	76 (8)	73 (8)	69 (7)	0
Asia	Dhaka	2014	4139	437 (11)	404 (10)	380 (9)	337 (8)	298 (7)	314 (8)	326 (8)	255 (6)	254 (6)	319 (8)	396 (10)	419 (10)	0
		2019	5084	485 (10)	453 (9)	458 (9)	419 (8)	390 (8)	419 (8)	398 (8)	346 (7)	389 (8)	407 (8)	421 (8)	499 (10)	0
	Kathmandu	2014	90	12 (13)	7 (8)	10 (11)	10 (11)	4 (4)	5 (6)	5 (6)	9 (10)	1 (1)	7 (8)	2 (2)	18 (20)	0
		2019	623	88 (14)	52 (8)	57 (9)	39 (6)	48 (8)	49 (8)	32 (5)	24 (4)	27 (4)	40 (6)	53 (9)	114 (18)	0
Latin America	Sao Paulo	2014	202	22 (11)	17 (8)	16 (8)	21 (10)	11 (5)	10 (5)	20 (10)	15 (7)	15 (7)	16 (8)	15 (7)	24 (12)	0
		2019	183	20 (11)	15 (8)	18 (10)	15 (8)	12 (7)	16 (9)	14 (8)	14 (8)	10 (5)	13 (7)	18 (10)	18 (10)	0
	Guadalajara	2014	325	35 (11)	37 (11)	22 (7)	20 (6)	31 (10)	14 (4)	19 (6)	35 (11)	33 (10)	17 (5)	27 (8)	32 (10)	3 (1)
		2019	319	40 (12)	31 (10)	19 (6)	17 (5)	19 (6)	18 (6)	12 (4)	31 (10)	23 (7)	25 (8)	34 (11)	36 (11)	14 (4)

The percentage of patients with a burn of more than 20% TBSA ranged from 19% to 81% across the centers. In Johannesburg, only 19% and 21% of burn admissions had a TBSA of more than 20% in 2014 and 2019, respectively, while in Sao Paulo 73% and 81% of burn admissions had a TBSA of more than 20% in 2014 and 2019, respectively. In most centers, the TBSA of more than 20% stayed similar between 2014 and 2019, apart from in Cairo where it dropped from 58% to 31%, and Ibadan where it dropped from 66% to 43%.

### Demographics of Burn Admissions Patients

For all centers with the sex of burn admissions patients reported, the percentage of men admitted was around 20% higher than women’s admissions (about 60% men and 40% women) (Table 3). In all centers apart from Nairobi, the percentage of male burn admissions increased slightly from 2014 to 2019. The distribution of burns admissions across adults and children varied widely across the centers. The percentage of adult burns admissions was much higher than children in Dhaka (72% in 2014, 79% in 2019), Kathmandu (76% in 2014, 74% in 2019), and Sao Paulo (88% in 2014, 85% in 2019), while the percentage of children burn admissions was much higher than adults in Guadalajara (75% in 2014, 78% in 2019), Johannesburg (68% in 2014, 64% in 2019), and Nairobi (56% in 2014, 57% in 2019). Most cities had a similar distribution of adult and children burn admissions between 2014 and 2019 apart from in Cairo and Ibadan where the majority of burn admissions changed from adults in 2014 to children in 2019.

**Table 3.** Sex and age group of burn admissions by center and year

				Sex			Age group		
Region	Center	Year	Total Admissions	Men	Women	Unknown	Children	Adults	Unknown
Africa	Cairo	2014	327	206 (63)	121 (37)	0	124 (38)	203 (62)	0
		2019	303	203 (67)	100 (33)	0	172 (57)	131 (43)	0
	Nairobi	2014	889	528 (59)	361 (41)	0	497 (56)	340 (38)	52 (6)
		2019	935	531 (57)	404 (43)	0	533 (57)	344 (37)	58 (6)
	Ibadan	2014	53	30 (57)	21 (39)	2 (4)	16 (30)	34 (64)	3 (6)
		2019	81	49 (60)	32 (40)	0	41 (51)	40 (49)	0
	Johannesburg	2014	882	562 (64)	320 (36)	0	596 (68)	286 (32)	0
		2019	909	618 (68)	291 (32)	0	582 (64)	327 (26)	0
Asia	Dhaka	2014	4139	2527 (61)	1612 (39)	0	1153 (28)	2986 (72)	0
		2019	5084	3305 (65)	1779 (35)	0	1083 (21)	4001 (79)	0
	Kathmandu	2014	90	5 (6)	20 (22)	65(72)	22 (24)	68 (76)	0
		2019	623	76 (12)	132 (21)	419 (67)	162 (26)	461 (74)	0
Latin America	Sao Paulo*	2014	202				25 (12)	177 (88)	0
		2019	183				27 (15)	156 (85)	0
	Guadalajara	2014	325	202 (62)	123 (38)	0	245 (75)	80 (25)	0
		2019	319	215 (67)^†^	113 (35)^†^	0	248 (78)	71 (22)	0

*Sex unknown for admissions.

^†^9 extra admissions for sex in Guadalajara 2019, adds up to 325 instead of 319 admissions.

### Location and Settings

The majority of burns took place in an urban location in Nairobi (81% in 2014, 81% in 2019), Guadalajara (83% in 2014, 76% in 2019), Cairo (79% in 2014, 94% in 2019), and Ibadan (68% in 2014, 88% in 2019), while the majority of burns took place in a rural location in Dhaka (67% in 2014, 79% in 2019) (Table 4). In all centers with available data on the setting of burn admissions, household burns were most common (36% to 79%). In Dhaka and Kathmandu, occupational burns were also common, 32% and 37%, respectively. In Dhaka, burns also took place in settings other than household and occupational settings (24% in 2014 and 27% in 2019).

**Table 4.** Location and settings of burn admissions by center and year

				Location			Setting			
Region	Center	Year	Total Admissions	Urban	Rural	Unknown	Household	Occupational	Other	Unknown
Africa	Cairo	2014	327	258 (79)	69 (21)	0	260 (79)	47 (14)	15 (5)	5 (2)
		2019	303	284 (94)	19 (6)	0	224 (74)	25 (8)	18 (6)	36 (12)
	Nairobi	2014	889	722 (81)	83 (9)	84 (10)	479 (54)	121 (14)	98 (11)	191 (21)
		2019	935	754 (81)	95 (10)	86 (9)	658 (70)	114 (12)	60 (7)	103 (11)
	Ibadan	2014	53	36 (68)	9 (17)	8 (15)	23 (43)	8 (15)	11 (21)	11 (21)
		2019	81	71 (88)	10 (12)	0	60 (74)	9 (11)	11 (14)	1 (1)
	Johannesburg^†^	2014	882	19 (2)	863 (98)	0				
		2019	909	30 (3)	879 (97)	0				
Asia	Dhaka	2014	4139	1366 (33)	2773 (67)	0	1697 (41)	1241 (30)	993 (24)	208 (5)
		2019	5084	1067 (21)	4017 (79)	0	1830 (36)	1677 (33)	1372(27)	205 (4)
	Kathmandu*	2014	90				63 (70)	27 (30)	0	0
		2019	623				346 (56)	277 (44)	0	0
Latin America	Sao Paulo*^†^	2014	202							
		2019	183							
	Guadalajara	2014	325	269 (83)	56 (17)	0	247 (76)	18 (6)	5 (1)	55 (17)
		2019	319	243 (76)	76 (24)	0	238 (75)	30 (9)	47 (15)	4 (1)

* Location of burn admissions not available.

^†^ Setting of burn admissions not available.

### Type of Burn Admissions

The most common type of burns across all centers in 2014 and 2019 were hot liquid and flame burns (Table 5). In most centers, the percentage of burns types was similar between 2014 and 2019. In Nairobi and Guadalajara, hot liquid burns were most common, followed by flame burns. In Cairo and Ibadan, the most common cause of burns was flame and hot liquid. In Dhaka, the types of burns were mostly flame (32% in 2014, 25% in 2019), hot liquid (28% in 2014, 32% in 2019), and direct electric burns (28% in 2014, 33% in 2019). In Sao Paulo, flame burns (45% in 2014, 44% in 2019) were most common followed by hot liquid (20% in 2014, 24% in 2019) and then direct electric burns (13% in 2014, 17% in 2019).

**Table 5.** Type of burn admissions by center and year

				Type of Burn							Type of Flame Burn*			
Region	Center	Year	Total Admission	Flame*	Hot Surface	Hot Liquid^†^	Direct Electric	Chemical	Other	Unknown	LPG	Kerosene	Solid Fuel	Unknown
Africa	Cairo	2014	327	173 (53)	3 (1)	124 (38)	12 (4)	15 (5)	0	0	138 (80)	27 (15)	0	8 (5)
		2019	303	101 (33)	0	133 (44)	15 (5)	18 (6)	0	36 (12)	80 (79)	7 (7)	0	14 (14)
	Nairobi	2014	889	184 (21)	14 (1)	417 (47)	58 (6)	7 (1)	44 (5)	165 (19)	18 (10)	133 (72)	0	33 (18)
		2019	935	253 (27)	21 (2)	459 (49)	63 (7)	9 (1)	47 (5)	83 (9)	88 (35)	132 (52)	7 (3)	26 (10)
	Ibadan	2014	53	29 (55)	0	10 (19)	1 (2)	1 (2)	0	12 (23)	2 (7)	6 (21)	18 (62)	3 (10)
		2019	81	32 (40)	1 (1)	33 (41)	9 (11)	0	3 (4)	3 (4)	10 (31)	2 (6)	19 (60)	1 (3)
	Johannesburg	2014	882	98 (11)	0	588 (67)	67 (8)	17 (2)	7 (1)	105 (12)	28 (29)	12 (12)	0	58 (59)
		2019	909	130 (14)	0	592 (65)	34 (4)	9 (1)	2 (0)	142 (16)	16 (12)	9 (7)	0	105 (81)
Asia	Dhaka	2014	4139	1324 (32)	372 (9)	1159 (28)	1145 (28)	90 (2)	49 (1)	0	131 (10)	36 (3)	1018 (77)	139 (10)
		2019	5084	1271 (25)	305 (6)	1626 (32)	1687 (33)	16 (0)	188 (4)	0	937 (74)	8 (1)	87 (6)	239 (19)
	Kathmandu^§^	2014	90	0	0	19 (21)	12 (13)	1 (1)	0	58 (64)				
		2019	623	0	7 (1)	144 (23)	69 (11)	11 (2)	0	392 (63)				
Latin America	Sao Paulo^§^	2014	202	91 (45)	12 (6)	40 (20)	27 (13)	4 (2)	17 (8)	11 (5)				
		2019	183	81 (44)	15 (8)	44 (24)	31 (17)	7 (4)	3 (2)	2 (1)				
	Guadalajara	2014	325	82 (25)	7 (2)	161 (50)	25 (8)	0	1 (0)	49 (15)	5 (6)	1 (1)	6 (7)	70 (86)
		2019	319	107 (34)^‡^	6 (2)^‡^	181 (57)^‡^	20 (6)^‡^	4 (1)^‡^	0^‡^	2 (0)^‡^	11 (10)	0	11 (10)	85 (80)

* Flame burns separated into subcategories.

^†^Hot liquid, steam, or gas.

^‡^ 9 extra admissions for type of burn in Dhaka 2019, adds up to 5093 instead of 5084 admissions.

^§^ Type of flame burn not available.

The type of burn flames varied largely depending on the center and year of burn admissions (Table 4). In Nairobi, the type of flame burn was mainly Kerosene in 2014 (72%), reducing in 2019 (52%), while the proportion due to LPG increased from 10% in 2014 to 35% in 2019. In Dhaka, the majority of flame burns changed from solid fuel (77%) in 2014 to LPG (74%) in 2019. In Cairo, the majority of flame burns were LPG regardless of the year (80% in 2014, 79% in 2019) followed by Kerosene (15% in 2014, 7% in 2019). In Ibadan, the majority of flame burns were solid fuel (62% in 2014 and 60% in 2019). The next most common type of flame burn was Kerosene in 2014 and LPG in 2019. The type of fuel associated with flame burns is either not recorded or mostly unknown in the other counties. Nevertheless, the rise in the proportion of flame burns due to LPG is a consistent finding among countries where data on fuel was recorded aligning in the switch from either solid fuel or kerosene to LPG seen across LMIC.

### Intentional or Nonintentional Burns

In all centers, the majority of burn admissions were due to unintentional burns (63–97%) (Table 6). In Nairobi, a substantial proportion of burns were caused by assault (22% in 2014, 20% in 2019) and intentional self-harm (10% in 2014, 12% in 2019). Differences between 2014 and 2019 in the cause of burns were limited due to missing information (in Guadalajara, Johannesburg, and Ibadan).

**Table 6.** Intentional vs nonintentional burn admissions by center and year

				Cause of burn			
Region	Center	Year	Total Admissions	Unintentional	Intentional Self-harm	Assault	Unknown
Africa	Cairo	2014	327	307 (94)	5 (2)	15 (5)	0
		2019	303	277 (91)	8 (3)	18 (6)	0
	Nairobi	2014	889	560 (63)	90 (10)	195 (22)	44 (5)
		2019	935	608 (65)	112 (12)	187 (20)	28 (3)
	Ibadan	2014	53	40 (75)	1 (2)	1 (2)	11 (21)
		2019	81	78 (97)	1 (1)	1 (1)	1 (1)
	Johannesburg	2014	882	820 (93)	4 (0)	58 (7)	0
		2019	909	582 (64)	0	0	327 (36)
Asia	Dhaka	2014	4139	4014 (97)	54 (1)	71 (2)	0
		2019	5084	4715 (93)	166 (3)	203 (4)	0
	Kathmandu	2014	90	0	0	0	90 (100)
		2019	623	605 (97)	16 (3)	2 (0)	0
Latin America	Sao Paulo*	2014	202				
		2019	183				
	Guadalajara	2014	325	258 (79)	8 (3)	3 (1)	56 (17)
		2019	319	295 (93)	14 (4)	7 (2)	3 (1)

* Cause of burn not available.

### Outcome of Burn Admissions

The outcomes of burn admissions by center and year were reported (Table 7). The mean length of stay in hospitals reduced in all centers from 2014 to 2019. The range of mean values varied from 11 to 33 days (median from 6 days to 26 days). In Nairobi and Dhaka, the average length of hospital stay is higher compared to the other centers. The percentage of deaths across the centers ranged from 5% to 42%. In Guadalajara, Johannesburg, and Sao Paulo, less than 10% of burn admission patients died. In Cairo and Ibadan, the percentage of deaths from burns admissions had dropped from 2014 to 2019 (7 and 20 percentage points, respectively).

**Table 7.** Outcomes of burn admissions by center and year

				Length of Stay in Hospital (Days)		
Region	Center	Year	Total Admissions	Mean	Median	Deaths
Africa	Cairo	2014	327	15	10	58 (18)
		2019	303	11	6	34 (11)
	Nairobi	2014	889	33	26	177 (20)
		2019	935	30	25	176 (19)
	Ibadan	2014	53	12	10	22 (42)
		2019	81	11	7	18 (22)
	Johannesburg*	2014	882			66 (7)
		2019	909			69 (8)
Asia	Dhaka	2014	4139	28	17	709 (17)
		2019	5084	22	15	937 (18)
	Kathmandu	2014	90	17	10	19 (21)
		2019	623	11	9	134 (22)
Latin America	Sao Paulo*	2014	202			15 (7)
		2019	183			15 (8)
	Guadalajara	2014	325	15		15 (5)
		2019	319	14		21 (7)

* Length of stay not available.

## DISCUSSION

As stated in the introduction, our data do not have denominator data which imposes a limitation on the inferences that we can draw. In addition, referral patterns likely changed during the course of the study so incident rate ratios may change as a result of a different spectrum of patients being admitted. For example, the proportion of children changed over time in some studies and children carry different risks of suffering burns of different causes. Thus, our data needs to be interpreted carefully and it is for this reason that we have not conducted statistical hypothesis tests. To add to the limitations of our study, not all centers collected the full WHO recommended list of items, for example recording the type of fuel associated with flame burns. What then can we infer from the data?

First, the data provides confirmation that burns remain a very serious problem in LMIC. The very observation that the Dhaka center treats more than 5000 severe burns in a year, of whom about 1000 die, is evidence that burn injuries are a very serious issue in LMIC.

Second, some changes are stark and are, arguably, unlikely to have resulted purely from selection effects. Thus, we find evidence in the data that a switch to LPG, from either kerosene or biomass, is associated with two outcomes. First, the total number of flame injuries decreases. Second, the proportion of flame injuries that are due to LPG increases. These findings suggest that the switch from one fuel to another may introduce new hazards even if the overall risk decreases. Ideally, the switch from one fuel to another should be accompanied by measures, both education and environmental modification, to mitigate the new risk. While biomass and kerosene fuels are inherently risky to use, LPG if used properly should essentially be risk-free for burns. If cases are occurring, then the fuel is being improperly used and prevention efforts are urgently needed.

Third, the spectrum of burn types across the different centers is very large and again would appear to exceed what might be attributed to selection effects alone. As we have seen, the proportion of burns associated with different fuels types is variable. The two African centers, Nairobi and Johannesburg, register the highest proportions of injury due to “hot liquids”; in essence hot/boiling water. The seasonal effect on burn injuries is most marked in Kathmandu and is likely caused by use of electrical heaters during the short but sharp winter. Perhaps the most provocative finding with respect to geographic variation is the very high proportion of intentional burn injuries in Nairobi.

Our data show there is no ‘one size fits all’ solution when it comes to intervention design. Just as different cities experience a different spectrum of pollutants, so to do cities have different predominant causes of burns. While the intervention must be tailored to the main cause of local burns, the cause specific solutions developed in one city should inform those in another. What is needed is evaluation of interventions that might reduce burn incidence so that cities can learn from each other. For example, local authorities in Nairobi can benefit from a study showing that the risk of immolation can be reduced by a public education campaign.^18^

Perhaps the largest implication of our findings, relate to the importance of better data collection regarding burns, especially severe burns, which have a high mortality and lifelong consequences. We hope that our findings can prompt renewed efforts to establish comprehensive burn registries. The WHO global burn registry form would enable burns units to collect data in a standardised way. It is the result of several years of collaboration lead by the WHO. It has been piloted to ensure that it is useable across a wide variety of settings and collects data on the epidemiology of burns along with clinical data. Widespread use of the WHO form would have overcome problems associated with the lack of standardisation that we encountered in this study. This in turn would provide a much more solid basis for initiatives to reduce burn injury. We therefore urge individual centers to adopt this tool that has been widely endorsed and that could be implemented at little to no marginal cost. We advocate for burn societies, public health agencies, and organisation of the United Nations, such as WHO, to vigorously promote use of the form. A registry or study based on the global burn registry would be limited to severe burns. We acknowledge that certain smaller burns, which do not prompt admission to a burn’s unit nevertheless have severe consequences. The number of facilities to which smaller burns may be reported is vast and record keeping in these disparate units is of variable quality. However, a number of studies have been conducted in which surveys have been successfully used to elicit population-based incidence data.^19–21^ Surveys are expensive and including burns data within wider registries may be a cost-effective way to harvest epidemiological data on burns in a regular basis.

In conclusion, our data provide important information on the causes of severe burns in LMIC and on how they vary by location. The findings show that burns remain a pressing issue even if we cannot provide a good numerical answer to the question of burn incidence and the data provide strong guidance in how to approach the development of burn injury prevention programs in LMIC.

## Supplementary Material

irac096_suppl_Supplementary_Appendix_1

## Notes

*Funding. None declared*.
