# Supplementary material for: Burn Admissions Across Low- and Middle-income Countries: A Repeated Cross-sectional Survey
Source: J Burn Care Res. 2022 Jul 8;44(2):320–8. doi: 10.1093/jbcr/irac096 (PMC9981866; doi:10.1093/jbcr/irac096)
Supplement: irac096_suppl_Supplementary_Appendix_1 [file irac096_suppl_supplementary_appendix_1.pdf]

World Health Organization

Global Burn Registry Data Collection Form

Date electronically entered: __________
(To be completed only by staff entering data electronically)

1. Indicate the respondent (person providing the information) and fill out all relevant boxes providing information about the patient.

Respondent	Patient's date of birth:	*If date of birth unknown:*	If under 5 years of age was the patient:	Sex:	Date of admission:	Hour of day admission:
Patient ☐ Husband ☐ Wife ☐ Parent ☐ Sibling ☐ Other ☐	__/__/____ *(Use dd/mm/yyyy format)*	Age in years: ____ *(Rounded to the nearest year)*	Alone ☐ With an adult but unsupervised ☐ With an adult and supervised ☐ With other children (<18 years old) ☐ Unknown ☐	Male ☐ Female ☐	__/__/____ *(Use dd/mm/yyyy format)*	______ *(use 24 hour clock – e.g. 15h not 3 p.m.)*

2. Fill out all relevant boxes indicating general and clinical information about the burn.

Date burn occurred:	Hour of day burn occurred:	Village, neighbourhood or postal code where burn occurred:	Total body surface area of burn *(refer to body surface area diagrams)*	Associated smoke inhalation injury	Associated injuries (check all that apply)
__/__/____ *(Use dd/mm/yyyy format)*	______ *(use 24 hour clock – e.g. 15h not 3 p.m.)*	______ *(Please use most specific locale. Postal code is best if available.)*	______ *(To nearest 5 % - e.g. 5%, 15%, 65%, etc.)*	Yes ☐ No ☐	No associated injuries ☐ Abdominal trauma ☐ Chest trauma ☐ Eye injury ☐ Long bone fracture ☐ Spinal cord injury ☐ Traumatic brain injury ☐

3. Fill out all relevant boxes indicating the anatomy of the burn (check all that apply).

Head and neck	Trunk	Arms	Hands and wrists	Legs
None ☐ Scalp ☐ Face ☐ Eye ☐ Neck ☐	None ☐ Chest, abdomen, back or buttocks ☐ Perineum or genitals ☐	None ☐ Shoulder and/or axilla ☐ Upper arm and/or forearm ☐ Elbow ☐	None ☐ Wrist ☐ Back of hand ☐ Palm ☐ Fingers and/or thumb ☐	None ☐ Thigh and/or lower leg ☐ Knee ☐ Ankle ☐ Foot ☐

4. Tick the appropriate box in the top row indicating how the burn was caused and then fill out the appropriate column below:

Flame ☐ *Occurring in which setting:*			Hot surface ☐ *Related to(select one best response from below):*	Hot liquid, steam or gas ☐ *Related to (select best response from below):*	Electrical ☐ *Occurring in which setting:*	Chemical ☐ *Occurring in which setting:*	Friction ☐ Inhalation ☐ Cooling ☐ Radiation ☐ Other ☐
Household ☐ *Related to (select one best response from below):*	Occupational ☐ *Type of occupation (select one best response from below):*	Public ☐ *Related to (select one best response from below):*	Cooking ☐ Household heating ☐ Household appliance ☐ Household lighting ☐ Occupational activity ☐ Other ☐	Cooking ☐ Bathing/washing ☐ Occupational activity ☐ Other ☐	Household ☐ Occupational ☐ Public ☐ *Related to:*	Household ☐ Occupational ☐ Public ☐	
Cooking ☐ Heating ☐ Lighting ☐ House fire (single) ☐ House fire (multiple) ☐ Intentional flame burn ☐ Playing with fire ☐ Other ☐	Food preparation ☐ Petrochemical ☐ Textiles ☐ Construction ☐ Agricultural ☐ Fireworks/related ☐ General industry ☐ Other ☐	Road traffic crash ☐ Bonfires ☐ Fireworks ☐ Spilled liquids ☐ Playing with fire ☐ Assault ☐ Terrorism or war ☐ Other ☐			High voltage ☐ Low voltage (<1,000 volts) ☐ Lightning ☐ Other ☐		

5. For burns involving either cooking/food preparation, household lighting, or household heating fill out all relevant boxes. For other burns, skip to question 6.

Cooking/food preparation		Household lighting	Household heating
Burn caused by contact with:	*Burn involved:*	*Burn caused by:*	*Burn caused by:*
Cook stove ☐ Cooking tool/vessel (pot, etc.) ☐ Burning fuel (wood, kerosene etc.) ☐ Cooked food or liquid ☐ Other ☐	Deliberate movement (e.g. deliberate touch) ☐ Accidental movement (e.g. fall/spill etc.) ☐ Explosion ☐ Fire in cooking area ☐ Other ☐	Lamp/lantern igniting surrounding material ☐ Deliberate movement touching lamp/lantern ☐ Accidental movement touching lamp/lantern ☐ Other ☐	Heating source igniting surrounding material ☐ Deliberate movement touching heating source ☐ Accidental movement touching heating source ☐ Other ☐
Details of cooking area:	*Fuel used for cooking:*	*Type of lamp/lantern:*	*Energy source for heating:*
Cooking area height (to ground): Under 0.9 m (3 feet) ☐ 0.9 m (3 feet) or higher ☐ Unknown ☐ *Cooking and living areas separate?* Yes ☐ No ☐	Ethanol ☐ Kerosene (Paraffin) ☐ Liquefied Petroleum Gas (LPG) ☐ Solar power ☐ Electricity ☐ Wood ☐ Charcoal ☐ Dung ☐ Coal ☐	Candle ☐ Kerosene (Paraffin) ☐ Electric ☐ Other ☐	Traditional biomass (wood, charcoal, dung) ☐ Coal ☐ Kerosene (Paraffin) ☐ Liquefied Petroleum Gas (LPG) ☐ Natural gas ☐ Electricity ☐ Other ☐

6. Burn caused intentionally? Intentional self-harm☐ Assault ☐ Unintentional☐ Undetermined intent☐

7. If "Undetermined intent" was selected above, what is the degree of clinical suspicion that the burn was caused intentionally?

None ☐ Low ☐ Medium ☐ High ☐

8. If the patient is 15 years or older, is the patient literate? Yes☐ No☐ Unknown☐

9. Contributing factors: None☐ Alcohol☐ Drug☐ Epilepsy☐ Dementia☐ Psychiatric illness☐ Physical or mental disability☐ Other☐

10. Number of people burned in this incident: 1 person☐ 2 people☐ 3-5 people☐ 6-9 people ☐ 10 and more people☐

11. Indicate the patients treatment and discharge.

Surgery during this hospital stay?	Date of discharge:	Hour of day patient discharged:	Condition on discharge from facility:
Yes ☐ No ☐	__/__/____ *(Use dd/mm/yyyy format)*	______ *(use 24 hour clock – e.g. 15h not 3 p.m.)*	Dead ☐ Transferred to another facility ☐ Left against medical advice ☐ Discharged home with disability ☐ Discharged home without disability ☐ Unknown ☐
